# Sacrococcygeal Teratoma associated with Trisomy 13

**DOI:** 10.21699/ajcr.v7i3.423

**Published:** 2016-06-15

**Authors:** Bayram Ali Dorum, Nilgün Köksal, Hilal Özkan, Sabahattin Karakaya, Ahsen Karagözlü Akgül

**Affiliations:** 1 Department of Pediatrics, Division of Neonatology, Faculty of Medicine, Uludag University, Bursa, Turkey; 2 Department of Pediatrics, Faculty of Medicine, Uludag University, Bursa, Turkey; 3 Department of Pediatric Surgery, Faculty of Medicine, Uludag University, Bursa, Turkey

**Keywords:** Chromosomal anomaly, Sacrococcygeal teratoma, Trisomy 13

## Abstract

Sacrococcygeal teratoma (SCT) is rarely associated with syndromes. We report a female newborn with a prenatal diagnosis of small sacrococcygeal teratoma and postnatally diagnosed as having trisomy 13. The sacrococcygeal teratoma was excised. It was reported as mature teratoma. The child succumbed to sepsis postoperatively.

## CASE REPORT

A female newborn delivered to a primigravida at 36th week of gestation via cesarean section because of fetal distress, had 1st and 5th min Apgar scores of 4/10 and 6/10 respectively. She was intubated because of poor spontaneous respiration. Antenatally, fetal MRI detected a mass in lower back as SCT. On physical examination, her birth weight was 2630g, height 40cm, and head circumference 30cm. Other abnormal findings included scaphocephaly, two occipitoparietal lesions measuring 14 and 16cm in diameter which were defined as aplasia cutis, microphthalmia, low-set ears, depressed nasal root, and polydactyly (Fig. 1). Aplasia cutis did not show any healing during hospitalization. The baby had a palpable subcutaneous sacral mass extending to the anal region measuring 43 mm x 55 mm (Fig. 2). Ventilatory support, fluid administration, and antibiotics were started. Complete blood picture revealed presence of thrombocytopenia, while other biochemical and coagulation tests were within normal limits. Abdominal ultrasound disclosed multiple renal cysts. Transfontanel ultrasound [USG] was unremarkable. On echocardiogram; a wide patent ductus arteriosus (PDA), polyvalvular myxomatous degeneration, and prolapse of atrioventricular valves were detected. On the 3rd day of hospitalization, sacral mass was excised. Histopathological diagnosis of the mass was reported as mature cystic teratoma. During the follow-up, hypoglycemic episodes and hyperinsulinism were detected. Despite glucose infusion at a rate of 12 mg/kg/min, normoglycemia could not be achieved, therefore diazoxide treatment was initiated. Cytogenetic analysis of the case was reported as trisomy-13. During the follow-up, multiple organ failure developed because of sepsis. Despite all supportive treatments, the infant died on postnatal day 18.

**Figure:**
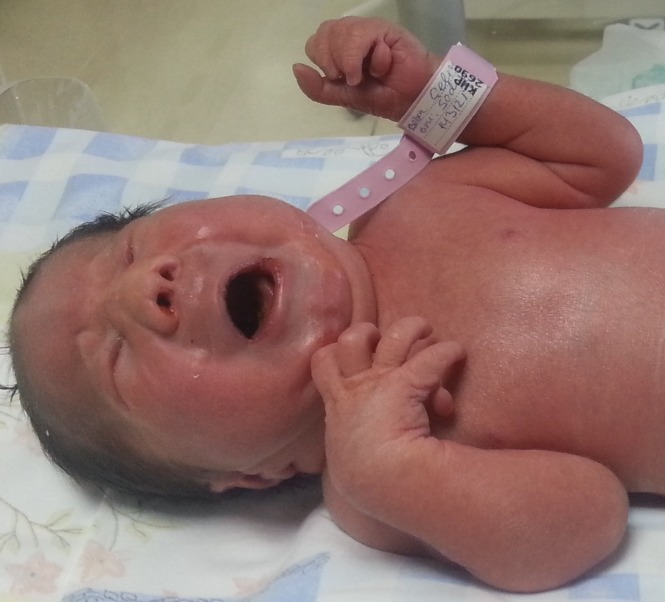
Figure 1:Polydactyly, microphthalmia, low-set ears, and depressed nasal root observed in our patient.

**Figure:**
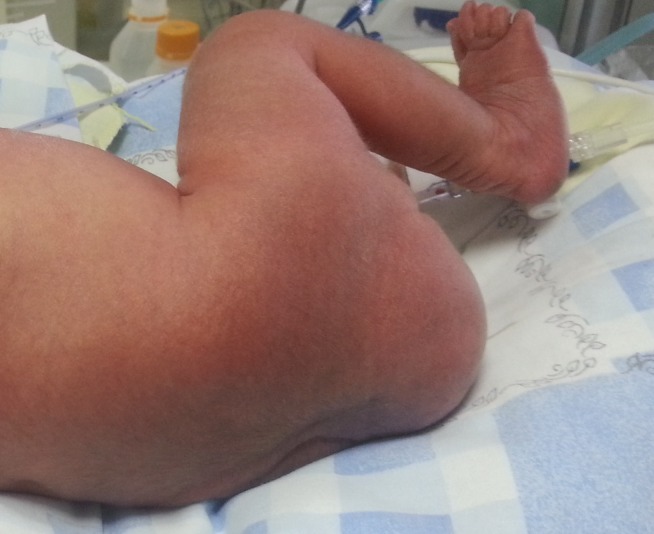
Figure 2:Small sacrococcygeal teratoma.

## DISCUSSION

SCT is rarely reported in association with chromosomal abnormalities and syndromes. It has been reported in association with Sotos syndrome, Wiedemann-Beckwith syndrome and Weaver syndrome.[1,2] Various chromosomal abnormalities reported in association with SCT are of chromosomes 1, 3, 10, 12 and, 13.[3] Trisomy 13 is rarely reported with teratoma on literature search. Dische et al reported teratomas of neck and liver in a trisomy 13 patient.[4] A fetus with oral cavity teratoma and trisomy 13 has also been reported.[5] Hargitai et al detected an umbilical cord teratoma in a 17-week fetus with trisomy 13.[6] Only one case of SCT has been reported in a suspected case of trisomy 13 but unfortunately in this case definitive genetic confirmation could not be made.[3] In our patient association of SCT with trisomy-13 was confirmed on genetic testing. These five cases may suggest an association between development of teratoma and chromosomal abnormalities.

The most frequent anomalies reported in patients with trisomy 13 (Patau's Syndrome) are congenital cardiac defects (80%), central nervous system defects (75%), eye, lip-palate, and finger anomalies (70%). In one-third of the patients urogenital anomalies, intrauterine growth retardation (IUGR), oligo or polyhydramnios, and prematurity have been reported.[7] PDA, atrial and ventricular septal defects are most frequently encountered cardiac anomalies. In our patient, PDA was also detected together with polyvalvular myxomatous degeneration and prolapse of atrioventricular valves. Our patient had microphthalmia, anophthalmia, and polydactyly affecting fingers. Scalp aplasia cutis congenita is rarely encountered in this syndrome. Renal involvement has been reported in one-third of the patients.[7] In our case polycystic kidney was also found.

Hyperinsulinemic hypoglycemia can be encountered as one of the clinical manifestations of the patients with trisomy-13. Normoglycemia can be achieved with diazoxide and high doses of glucose infusion.[8] In our patient too high rate of insulin infusion and diazoxide were started to achieve normoglycemia, but she died because of sepsis.

## Footnotes

**Source of Support:** Nil

**Conflict of Interest:** None declared

